# The Effect of Intra-articular Hypertonic Dextrose Prolotherapy on Pain, Quality of Life, and Functional Outcomes Scores in Patients With Knee Osteoarthritis

**DOI:** 10.7759/cureus.48096

**Published:** 2023-11-01

**Authors:** Rock P Vomer, Rayghan S Larick, Ryan Milon, Emma York

**Affiliations:** 1 Family Medicine, Mayo Clinic Jacksonville Campus, Jacksonville, USA; 2 Family Medicine and Community Health, Duke University, Durham, USA; 3 Physical Medicine and Rehabilitation, Eastern Virginia Medical School, Norfolk, USA; 4 Sports Medicine, Mayo Clinic Jacksonville Campus, Jacksonville, USA; 5 Family Medicine, Prisma Health University of South Carolina, Columbia, USA

**Keywords:** quality of life (qol), functional improvement, chronic pain management, osteoarthritis (oa), prolotherapy

## Abstract

Introduction

Knee osteoarthritis is a common chronic condition leading to pain and debility. Prolotherapy is a safe and cost-effective treatment option. Our team conducted a case series to examine the effect of prolotherapy on functional measures and quality of life in patients with knee osteoarthritis.

Methods

Our case series included 15 total patients consisting of male and female patients aged 50-85 years old with a prior diagnosis of knee osteoarthritis and moderate pain. The patients completed baseline Western Ontario and McMaster Universities Arthritis Index (WOMAC) and EuroQol-5 Dimension (EQ5D) surveys and received an ultrasound-guided intra-articular prolotherapy injection. The patients were then followed up by Telehealth at one-, two-, and three-month post-procedure.

Results

Data points for 15 patients were recorded over the duration of the three months. Results revealed significantly improved functional scores from the baseline period (M = 0.60) to the one-month (M = 0.43), two-month (M = 0.48), and three-month (M = 0.46), observed power = 0.99. Participants reported significantly lower pain scores between the baseline period (M = 0.60) compared to the one-month (M = 0.34), two-month (M = 0.35) and three-month (M = 0.39) periods. There was no statistical significance across time in participants’ EQ5D scores. The repeated-measures analysis of variance (ANOVA) revealed no statistically significant differences in participants scores between the baseline (M = 64.4), one-month (M = 68.93), two-month (M = 68.47), or three-month (M = 68.33), observed power = 0.31. There were no statistically significant differences in participants WOMAC stiffness scores between the baseline (M = 0.67), one-month (M = 0.46), two-month (M = 0.57), or three-month (M = 0.56) periods, observed power = 0.73. The results revealed no significant change in participants’ EQ5D and WOMAC stiffness scores. The results support a statistically significant improvement in patients’ self-reported functioning and pain scores between the baseline and one-month, two-month, and three-month periods.

Discussion

The significant improvement in function occurred between the baseline and one-month time periods and the pain reduction was sustained throughout the two-month and three-month periods. Our study supports prolotherapy as an effective treatment option to improve pain and function in knee osteoarthritis. The study could be repeated with a large sample size to further investigate the effects of prolotherapy on quality-of-life measures.

## Introduction

Osteoarthritis (OA) of the knee is a common chronic condition that causes significant pain and decreased function [1]. Knee OA is the most common joint condition in the United States and is increasing in prevalence with the aging population in the setting of an obesity epidemic [2]. Pain from OA originates from a combination of intra- and extra-articular knee structures including the medial, lateral, and patellofemoral compartments and their bony, ligamentous, and cartilaginous components [2,3]. The pathophysiology of OA is poorly understood and remains under investigation but is thought to be multifactorial, originating from both chronic, low-grade inflammatory and biomechanical processes [2]. OA involves a variety of “wear and tear” joint changes including articular cartilage damage, bony osteophyte formation, sclerosis of the subchondral bone, and, when advanced, subchondral cyst formation [3]. Patients with knee OA most often complain of pain that is worse with activity, joint stiffness, and difficulty with purposeful movements. Diagnosis is usually made by history and physical exam and confirmed with x-rays.

Knee OA increases in prevalence as patients age and has become increasingly expensive to treat [4]. Knee OA has an estimated prevalence of 33.6% affecting 12.4 million individuals living in the US [2]. Most often care is a multidisciplinary approach focused on weight reduction, physical therapy, assistive device use, bracing, and medical management of pain symptoms [5]. There is a continual focus on developing cost-effective treatment and preventative options [5]. There are both non-surgical and surgical treatment options for knee OA, with surgery reserved for those who fail conversation treatments. Currently, there are various conservative procedural treatment options including intra- and extra-articular injections of corticosteroid, hyaluronic acid, platelet-rich plasma, prolotherapy, stem cells, as well as genicular nerve blocks [6].

Prolotherapy, which involves injection of hypertonic dextrose, is a minimally invasive and cost-effective regenerative treatment option for knee OA. Prolotherapy facilitates healing through tissue proliferation which is thought to be potentially mediated by an inflammatory mechanism [7,8]. This therapy typically utilizes single or multiple injections containing hypertonic dextrose, applied to both intra- and extra-articular structures. The multiple injection technique causes some level of discomfort to patients and requires advanced training not typically included in standard family medicine residency education [9]. Currently, there is a moderate level of evidence supporting prolotherapy usage as a standard of care regarding pain reduction and functional improvement, but the body of literature is small [10].

Providing an intra-articular prolotherapy injection to treat knee OA is safe and within the scope of primary care providers [11]. Given the level of safety and moderate level of evidence for treatment with prolotherapy, expanding the body of literature is beneficial to patients and providers alike. At present, prolotherapy is not a standard injection provided at our academic-based community family medicine clinic. Prolotherapy treatments have the potential to be a cost-effective and clinically beneficial treatment for patients. Consequently, we have decided to conduct a case series examining the effect of intra-articular prolotherapy on pain and functional outcome measures and quality of life scores in patients who have knee OA.

## Materials and methods

Study design

This research study was a case series of 15 patients. The patients were selected based on the inclusion criteria of being 50-80 years old, having a prior diagnosis of knee OA, and having moderate knee pain for at least three months. Patients were educated on the risks and benefits of prolotherapy. The patients were consented with formal written consent for treatment. Data for pain score and functional impairment were collected via Western Ontario and McMaster Universities Arthritis Index (WOMAC), and quality of life scores were collected via the EuroQol-5 Dimension (EQ5D). These data points were collected at baseline, one-, two- and three-month post-injection. The data at one two- and three-month post-injection were collected via a scripted phone call. 

WOMAC description

**Figure 1:**
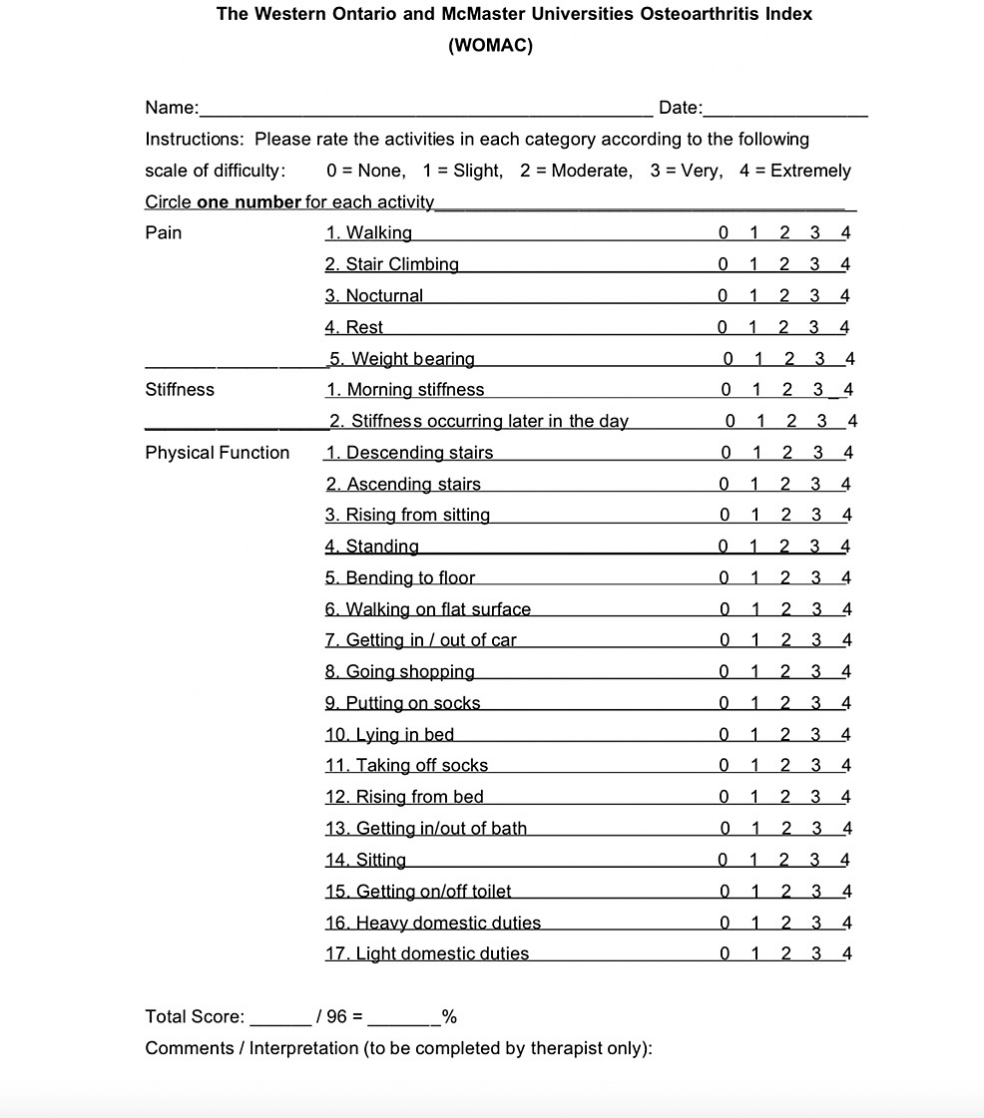
WOMAC Questionnaire

The WOMAC is a questionnaire that evaluates patient-reported pain, stiffness, and physical function, each section consisting of five, two, and 17 questions, respectively. Questions are rated on the Likert scale of 0-4, with lower scores indicating lower levels of pain, stiffness, and functional disability. Scores in each section are summed to produce a total summary index score. Higher index scores on WOMAC indicate worse pain, stiffness, and functional limitations (Figure 1) [12].

EQ5D description

**Figure 2:**
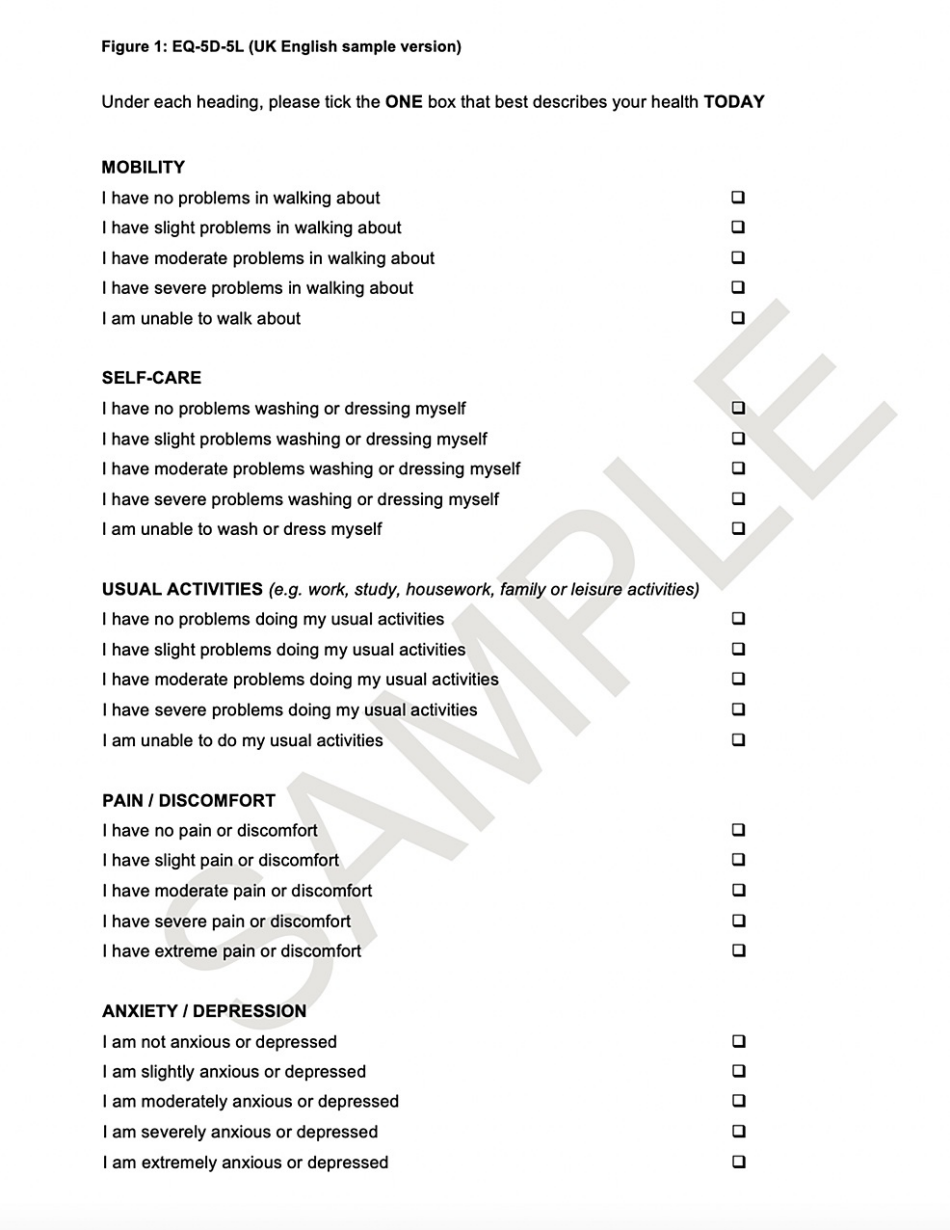
EQ5D Questionnaire

**Figure 3:**
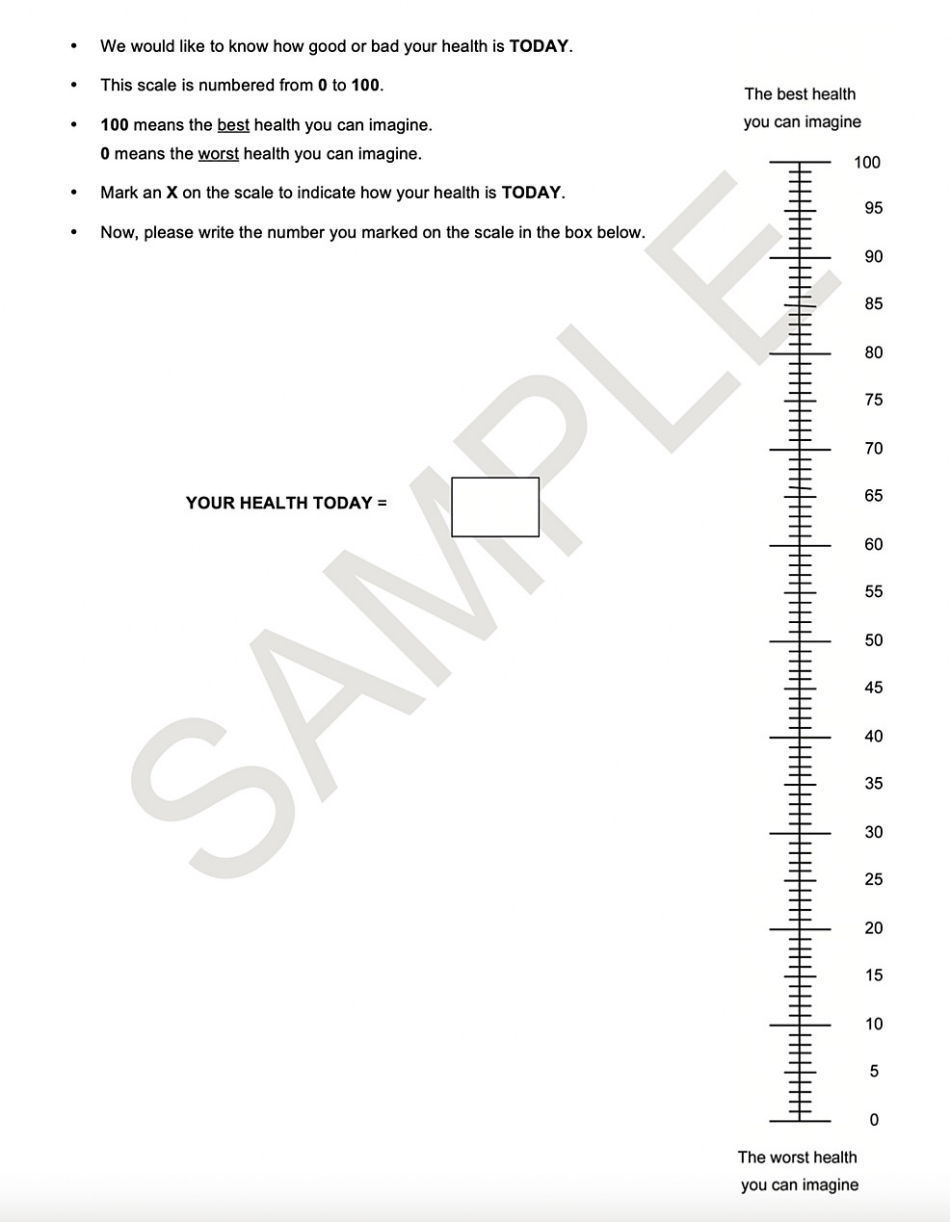
EQ5D health rating questionnaire

The EQ5D is a questionnaire that evaluates patients' quality of life. The EQ5D comprises five questions on mobility, self-care, pain, daily activities, and psychosocial status with three possible answers for each (1 = no problem, 2 = moderate problem, 3 = serve problem). The five items are summarized into a single summary index with a maximum score of 1, indicating the best health state. Additionally, the EQ5D includes a visual analog scale indicating general health status with a maximum score of 100, indicating best health status (Figures 2, 3) [13].

Data collection

Baseline data for the initial WOMAC and EQ5D scoring took place in person. For subsequent data point collection, the patients were contacted via phone call post-injection at one-, two- and three-month intervals to reassess WOMAC and EQ5D scores. All protected health information was maintained in strict confidence as required by law. Patients’ data was recorded and stored in a password-protected electronic database, in accordance with the standards of practice for research. The de-identified data set and the study ID key were kept separately in accordance with recommendations of the Office of Human Research Protections (OHRP) and the guidelines of Good Clinical Practice. Access to all patient records was restricted to authorized personnel only.

Data analysis

Descriptive statistics mean (96% confidence interval) or median (interquartile), min, max, standard error, or frequency were utilized to summarize the data [14]. A paired t-test was utilized to compare the WOMAC and EQ5D scores at one, two-, and three-month post-injection to their respective baseline scores pre-injection; If data are skewed, the variables will be transformed and made more normal, or the Wilcoxon signed-rank test will be utilized for the comparison [15]. For multiple comparisons, one-way repeated measures ANOVA, Friedman's test rank-based were utilized depending on the data [16]. To control the false positive rate Benjamini-Hochberg method was utilized for the adjustment of multiple comparisons [17].

The sample size of 20 achieves 81% power to detect a mean of paired differences of 0.7 with an estimated standard deviation of differences of 1.0 and with a significance level (alpha) of 0.05000 using a two-sided paired t-test. We assumed a dropout rate of 10%, then the recruited samples will be 15 samples [18,19].

## Results

Quality of life (EQ5D)

The categorical level independent variable (IV) Time was comprised of the following three levels: Baseline, one month, two months, and three months. The continuous level dependent variable was participants’ scores on the EQ5D scale. Based on the recommendations of Flinn and Kalkbrenner, a repeated-measures analysis of variance (ANOVA) is the most appropriate statistical test. Assumption checking was completed to ensure that the EQ5D data met the following statistical assumptions for a repeated-measures ANOVA: Normality of scores, absence of extreme outliers, and sphericity.

An investigation of standardized z-scores revealed an absence of outliers (z < + - 3.29; Field, 2018). Skewness and kurtosis values showed that the data were consistent with a normal distribution (skewness < + - 2 and kurtosis < + -7). Mauchly’s test of sphericity (W) revealed that the statistical assumption of sphericity was violated, X2(5) = 19.51, W = 0.22, p = 0.002. Accordingly, a Greenhouse-Geisser adjustment was applied. The repeated-measures ANOVA revealed no statistically significant differences in participants scores between the baseline (M = 64.4), one-month (M = 68.93), two-month (M = 68.47), or three-month (M = 68.33), F = (3, 25.66) = 1.70, p = 0.205, = 0.11, observed power = 0.31 (Table 1).

**Table 1:** Change in the quality-of-life scores on the EQ5D

Subject	Baseline	Month 1	Month 2	Month 3
1	80	83	83	85
2	60	70	80	75
3	60	70	72	72
4	60	70	75	80
5	70	70	75	78
6	10	25	35	35
7	60	65	60	60
8	75	50	55	55
9	90	95	90	90
10	93	95	95	90
11	50	69	50	50
12	88	90	90	85
13	50	55	50	50
14	60	60	50	55
15	60	67	67	65

Pain and functional impairment (WOMAC)

Consistent with the EQ5D data analysis, the WOMAC analysis included time as the IV with the same three levels (baseline, one month, two months, and three months). The continuous level dependent variable was participants’ scores on the Western Ontario and McMaster Universities Arthritis Index (WOMAC). The WOMAC is comprised of three interval-level subscales including pain, stiffness, and function. Three separate univariate ANOVAs were computed instead of a multivariate analysis due to statistical power considerations. A Bonferroni correction was applied to control for the familywise error rate (adjusted alpha = 0.013). An investigation of standardized z-scores for all three WOMAC scales revealed an absence of outliers (z < + - 3.29; Field, 2018). Skewness and kurtosis values for all three WOMAC scales indicated that the data were consistent with a normal distribution (skewness < + - 2 and kurtosis < + -7.

Participants' scores on the WOMAC pain scale were entered into the first ANOVA as the DV. Mauchly’s test of sphericity revealed that the statistical assumption of sphericity was violated, X2(5) = 0.36, W = 0.36, p = 0.022. As a result, a Greenhouse-Geisser adjustment was applied. Results revealed a statistically significant main effect for Time, F = (1.945, 42) = 11.21, p < 0.001, = 0.45, observed power = 0.99. Specifically, participants reported significantly lower pain scores between the baseline period (M = 0.60) compared to the one-month (M = 0.34), two-month (M = 0.35) and three-month (M = 0.39) periods (Tables 2-5).

**Table 2:** Subjects baseline WOMAC scores for pain, stiffness and function

Subject	Pain	Stiffness	Function	Total
1	16.00	3.00	37.00	56.00
2	16.00	6.00	51.00	73.00
3	13.00	6.00	55.00	74.00
4	18.00	8.00	50.00	76.00
5	13.00	8.00	50.00	71.00
6	10.00	5.00	33.00	48.00
7	13.00	6.00	34.00	53.00
8	12.00	5.00	27.00	44.00
9	4.00	2.00	25.00	31.00
10	2.00	2.00	9.00	13.00
11	15.00	8.00	68.00	91.00
12	1.00	2.00	7.00	10.00
13	14.00	5.00	50.00	69.00
14	15.00	7.00	55.00	77.00
15	17.00	7.00	60.00	84.00

**Table 3:** Subjects one-month WOMAC scores for pain, stiffness, and function

Subject	Pain	Stiffness	Function	Total score
1	10.00	4.00	32.00	46.00
2	7.00	5.00	44.00	56.00
3	4.00	6.00	38.00	48.00
4	5.00	4.00	45.00	54.00
5	3.00	2.00	12.00	17.00
6	2.00	3.00	18.00	23.00
7	12.00	5.00	27.00	44.00
8	13.00	5.00	23.00	41.00
9	3.00	2.00	21.00	26.00
10	1.00	1.00	7.00	9.00
11	2.00	1.00	21.00	24.00
12	0.00	2.00	3.00	5.00
13	12.00	4.00	43.00	59.00
14	14.00	6.00	54.00	74.00
15	15.00	5.00	54.00	74.00

**Table 4:** WOMAC scores two months after intervention for pain, stiffness, and function

Subject	Month Pain	Month Stiffness	Month Function	Total score
1	8.00	4.00	33.00	45.00
2	6.00	4.00	43.00	53.00
3	4.00	5.00	39.00	48.00
4	6.00	7.00	38.00	51.00
5	1.00	2.00	18.00	21.00
6	2.00	3.00	19.00	24.00
7	7.00	6.00	37.00	50.00
8	11.00	5.00	23.00	39.00
9	2.00	2.00	21.00	25.00
10	1.00	2.00	7.00	10.00
11	13.00	8.00	54.00	75.00
12	1.00	3.00	8.00	12.00
13	13.00	4.00	46.00	63.00
14	15.00	7.00	55.00	77.00
15	16.00	6.00	52.00	74.00

**Table 5:** WOMAC scores three months after intervention for pain, stiffness, and function

Subject	Pain	Stiffness	Function	Total score
1	15.00	5.00	36.00	56.00
2	8.00	4.00	42.00	54.00
3	3.00	6.00	39.00	48.00
4	6.00	6.00	32.00	44.00
5	1.00	2.00	19.00	22.00
6	2.00	2.00	20.00	24.00
7	9.00	6.00	29.00	44.00
8	12.00	5.00	24.00	41.00
9	3.00	2.00	24.00	29.00
10	2.00	2.00	9.00	13.00
11	9.00	7.00	34.00	50.00
12	1.00	2.00	7.00	10.00
13	14.00	5.00	47.00	66.00
14	16.00	7.00	55.00	78.00
15	17.00	6.00	52.00	75.00

Participants' scores on the WOMAC stiffness scale were entered into the second ANOVA as the DV. Mauchly’s test of sphericity revealed that the statistical assumption of sphericity was violated, X2(5) = 18.14, W = 0.24, p = 0.022. To this end, a Greenhouse-Geisser adjustment was applied. The repeated-measures ANOVA revealed no statistically significant differences in participants WOMAC stiffness scores between the baseline (M = 0.67), one-month (M = 0.46), two-month (M = 0.57), or three-month (M = 0.56) periods, F = (2.08, 42) = 4.47, p = 0.019 (adjusted alpha = 0.013) = 0.24, observed power = 0.73.

Participants' scores on the WOMAC function scale were entered into the second ANOVA as the DV. Mauchly’s test of sphericity revealed that the statistical assumption of sphericity was violated, X2(5) = 19.53, W = 0.22, p = 0.002. Accordingly, a Greenhouse-Geisser adjustment was applied. Results revealed a statistically significant main effect for Time, F = (1.62, 42) = 8.95, p = 0.003, = 0.38, observed power = 0.91. Specifically, participants reported significantly lower function scores between the baseline period (M = 0.60) compared to the one-month (M = 0.43), two-month (M = 0.48) and three-month (M = 0.46) periods.

## Discussion

Currently, there is a moderate level of evidence supporting prolotherapy usage as a standard of care regarding pain reduction and functional improvement, but the body of literature is small [10]. Prolotherapy has gained popularity as a form of regenerative therapy for knee OA. The exact mechanism by which prolotherapy exerts its regenerative effects is unclear. It is theorized that the chemical irritant, such as hypertonic dextrose, causes a localized pro-inflammatory response in peri- and intra-articular structures which stimulates growth factors and cytokines to promote healing in ligaments, tendons, and intra-articular cartilage [20]. Prolotherapy is contraindicated in patients with an allergy to the local anesthetic or other components of the injected solution and in patients with the following conditions: acute arthritis, active infection, complete tendon or ligament rupture, and rheumatoid arthritis [20]. Prolotherapy is generally well tolerated and does not impose significant side effects. Most commonly, patients experience increased pain for one to two days following the procedure [20]. As with any procedure, there is a rare risk of infection which is minimized by the use of sterile practices. Multiple systematic reviews and meta-analyses of the use of prolotherapy for the treatment of knee OA concluded that hypertonic dextrose prolotherapy appears to be a safe and effective treatment option with efficacy to improve pain and function in patients with knee OA [20-23]. The current body of literature has been unable to conclude the superiority of prolotherapy compared to other common intra-articular injection procedures such as corticosteroids, hyaluronic acid, platelet-rich plasma, erythropoietin, or stem cells [20-23].

The results presented in the current case series are in line with previous research and add to the growing body of literature regarding the safety and efficacy of prolotherapy for the reduction of pain and improvement of physical function [20-23]. We found a statistically significant reduction in participants’ self-reported pain between the baseline and one-month, two-month, and three-month periods. The effect size (i.e., practical significance) of this finding was in the strong range based on the guidelines provided by Sink and Mvududu [24]. This suggests that participation in prolotherapy was associated with a notable reduction in patients’ self-reported pain levels. Results also suggest that a significant reduction in pain occurred between the baseline and one-month time periods, and the pain reduction was sustained throughout the two-month and three-month periods. Additionally, we found a statistically significant reduction in patients’ self-reported functional disability scores between the baseline and one-month, two-month, and three-month periods. The effect size of this finding was in the strong range, indicating that participation in prolotherapy was associated with a notable reduction in functioning. Consistent with findings for the pain scale, the significant reduction in functioning seemed to occur between the baseline and one-month time periods, and the pain reduction was sustained throughout the two-month and three-month periods.

Results revealed an absence of statistical significance across time in participants’ quality of life EQ5D scores. Sit et al. conducted a single center, parallel group, blinded, randomized controlled trial in a primary care clinic in Hong Kong comparing intra-articular injection of prolotherapy vs. saline and found significant improvements in pain and function as measured via WOMAC and quality of life as measured via EQ5D [11]. It is possible that the small sample size (N = 15) contributed to our findings (i.e., type II error), as a retrospective power analysis revealed a low power estimate (.31). It is also possible that prolotherapy was not associated with a statistically significant reduction in participants’ EQ5D scores in our population. Future researchers should recruit a larger sample size to test these possible explanations for this finding. There was also an absence of statistically significant differences in participants’ self-reported stiffness on the WOMAC. However, the retrospective power analysis was less than the conventional .80 threshold. Accordingly, it is possible that a type II error occurred due to the small sample size. Future investigators should replicate this study with a larger sample size.

It is important to address the limitations of our current study. As we present a case series, there was no control group, so we are unable to determine if these results are due to prolotherapy alone. Our case series involved a small sample size of fifteen patients. Knee OA is a chronic condition, and it is unknown what the effects of prolotherapy are in our current population beyond the three-month follow-up.

This case series highlights the potential benefits of prolotherapy in improving patients' self-reported pain and function scores following intra-articular ultrasound-guided prolotherapy injection for knee OA in an academic-based family medicine clinic. This has important implications regarding the feasibility and utility of implementing prolotherapy as a standard treatment option in academic-based family medicine clinics where medical trainees learn this treatment approach as part of their primary care training. This case series serves as a contribution to the growing body of literature on the efficacy of prolotherapy. More robust research to investigate the feasibility, efficacy, and cost in comparison to other standard treatments already offered in most academic-based family medicine clinics is needed.

## Conclusions

This case series examined the safety and cost-effectiveness of prolotherapy as a treatment for knee OA. We found a statistically significant improvement in patients’ self-reported functioning and pain scores between the baseline and one-month, two-month, and three-month periods. A significant improvement in function occurred between the baseline and one-month time periods and the pain reduction was sustained throughout the two-month and three-month periods. Collectively, the results support prolotherapy as an efficacious treatment for knee OA by reducing pain and improving function. Further research with a larger sample size will be necessary to determine the effects of prolotherapy on quality-of-life measures and establish it as a safe and effective treatment for knee OA in the future.
